# Mutational spectrum of syndromic genes in sporadic brain arteriovenous malformation

**DOI:** 10.1186/s41016-022-00270-8

**Published:** 2022-02-24

**Authors:** Kun Wang, Mingqi Zhang, Sen Zhao, Zhixin Xie, Yisen Zhang, Jian Liu, Ying Zhang, Xinjian Yang, Nan Wu

**Affiliations:** 1 Department of Interventional Neuroradiology, Beijing Neurosurgical Institute and Beijing Tiantan Hospital, Capital Medical University, Beijing, 100050 China; 2 Department of Orthopedic Surgery, State Key Laboratory of Complex Severe and Rare Diseases, Peking Union Medical College Hospital, Peking Union Medical College and Chinese Academy of Medical Sciences, Beijing, 100730 China; 3 Beijing Key Laboratory for Genetic Research of Skeletal Deformity, Beijing, 100730 China; 4 Key Laboratory of Big Data for Spinal Deformities, Chinese Academy of Medical Sciences, Beijing, 100730 China

## Abstract

**Background:**

Brain arteriovenous malformations (BAVMs) are abnormal vessels that are apt to rupture, causing life-threatening intracranial hemorrhage (ICH). The estimated prevalence of BAVMs is 0.05% among otherwise healthy individuals. In this study, we aim to investigate the mutational spectrum of syndromic genes in sporadic BAVM.

**Methods:**

We recruited a cohort of 150 patients with BAVM and performed whole-exome sequencing on their peripheral blood DNA. To explore the mutational spectrum of syndromic genes in sporadic brain arteriovenous malformation, we selected six genes according to the Online Mendelian Inheritance in Man (OMIM) and literature. All variants in the six candidate genes were extracted and underwent filtering for qualifying variants.

**Results:**

There are a total of four patients with rare variants in hereditary hemorrhagic telangiectasia-related genes. In addition, we identified two patients have the variant of *RASA1* gene in our database, which are also rare mutations that are absent from population databases. However, we did not find any patients with *GNAQ* mutations in our database.

**Conclusions:**

In conclusion, we demonstrated that variants in syndromic vascular malformations play important roles in the etiology of sporadic BAVM.

## Background

Brain arteriovenous malformations (BAVMs) are abnormal vessels that are apt to rupture, causing life-threatening intracranial hemorrhage (ICH) [1]. The estimated prevalence of brain BAVMs is 0.05% among otherwise healthy individuals [2]. BAVMs account for 25% of hemorrhagic strokes in adults younger than 50 years of age, and up to 40% of BAVM patients die or remain functionally impaired within one year after ICH [3].

Although the pathogenesis of sporadic BAVM is largely unknown, some BAVM cases are associated with hereditary hemorrhagic telangiectasia (HHT) and capillary malformation-arteriovenous malformation (CM-AVM) [4, 5]. Moreover, rare congenital syndrome is present in featuring intracranial vascular malformations, such as Sturge-Weber syndrome [6]. Approximately 30% of affected syndromic individuals also have fast-flow AVM [7]. Life-threatening complications can arise from these fast-flow lesions including hemorrhage and neurological consequences requiring transarterial embolization or surgical treatment [8].

In recent years, whole exome sequencing (WES) has developed as a reliable technology for identifying coding mutations at a genome-wide level. WES makes it possible to identify predisposing variants for rare diseases such as BAVM. De novo variants in *PITPNM3*, *SARS*, and *LEMD3* have been identified in sporadic BAVM in our research previously [9]. We also identified some compound heterozygous mutations through BAVM probands and their healthy parents [10]. These results opened a new avenue to further explore the pathogenesis of BAVMs.

In this study, we selected six candidate genes related to the syndromic BAVM to investigate the mutational spectrum of syndromic genes in sporadic BAVM.

## Methods

### Patient recruitment

Sporadic cases were consecutively recruited into this study following the including criteria: (1) bAVM lesion confirmed by both a neurosurgeon and a neuroradiologist according. (2) With written informed consent from the proband or familial members.

Exclusion criteria were (1) known diagnoses of hereditary hemorrhagic telangiectasia, capillary malformation-AVM, Sturge-Weber syndrome, or other Mendelian vascular disorders; and (2) incomplete clinical data.

### Exome sequencing

Genome DNA was extracted from the peripheral blood for all individuals and available parental samples using DNeasy Blood Kits (QIAGEN, Eastwin Scientific, Inc. Beijing, China) according to the manufacturer’s instructions. SureSelect Human All Exon V6+UTRr2 core design was used for exome capture in cases and their parents. DNA sequencing was performed on Illumina HiSeq 4000 or Novaseq platform.

### Bioinformatic analysis

The variant-calling and annotation were performed by the in-house developed PUMP (Peking Union Medical college hospital Pipeline) [11, 12]. Single-nucleotide variants and internal duplications and/or deletions (indels) were called using the HaplotypeCaller of the Genome Analysis Toolkit, version 3.4.0. Annotated of the de novo, compound heterozygotes and recessive inherited variants were calculated with Gemini (version 0.19.1) for in silico subtraction of parental variants from the proband’s variants, with accounting for read number information extracted from BAM files. Computational prediction tools (GERP++ [13], CADD [14], SIFT [15], and Polyphen-2 [16]) were used to predict the conservation and pathogenicity of candidate variants. All variants were compared against publicly available databases such as the 1000 Genomes Project (http://www.internationalgenome.org/), the Exome variant server, NHLBI GO Exome Sequencing Project (ESP) (http://evs.gs.washington.edu/EVS/), and the genome aggregation database (gnomAD) (https://gnomad.broadinstitute.org/)

### Candidate gene selection

To explore the mutational spectrum of syndromic genes in sporadic bAVM, we selected six genes according to the Online Mendelian Inheritance in Man (OMIM) (https://www.omim.org/) and literature. The syndromes include hereditary hemorrhagic telangiectasia (HHT, OMIM #PS187300) caused by germline, heterozygous loss of function (LoF) variants in either *ENG* [17], *ACVRL1* [18], *BMPR2* [19], or *SMAD4* [20], capillary malformation-arteriovenous malformation (CM-AVM, OMIM #608354) which is caused by heterozygous LoF variants in *RASA1* [21], as well as in Sturge-Weber syndrome (OMIM #185300) which is caused by somatic activating mutations in *GNAQ* [22].

### Variant prioritization

All variants in the six candidate genes were extracted and underwent filtering for qualifying variants. We ask the variants to be either LoF variants (stop-gained, frameshift or canonical splice variants) or be deleterious missense varants/indels predicted to be deleterious (CADD score ≥ 15). Qualifying variants were also to have a minor allele frequency less than 0.001 in the gnomAD database.

## Results

### Cohort enrolment

A total of 150 probands with a clinical diagnosis of BAVM were consecutively enrolled between 2018 and 2020. Detailed clinical characteristics are presented in Table 1. Patients were independently reviewed by two experienced neuroradiologists to verify the diagnosis of BAVM by radiology imaging of the cerebrovascular system (magnetic resonance imaging/angiography (MRI/MRA), computed tomography angiography (CTA), and three-dimensional digital subtraction angiography (DSA)).

**Table 1:** Clinical characteristics of patients with BAVM

Characteristics	BAVM cohort
Total number of patients	150
Male, No. (%)	78 (52.0)
Age of diagnosis, mean (SD), year	14.2 (6.84)
Under 18 year, No. (%)	83 (55.3)
Main symptom	
Hemorrhage, No. (%)	86 (57.3)
Headache, No. (%)	18 (12.0)
Seizure, No. (%)	21 (14.0)
Focal neurologic deficit, No. (%)	15 (10.0)
Asymptomatic, No. (%)	11 (7.3)
BAVM location	
Temporal	34 (22.7)
Basal ganglia	21 (14.0)
Frontal	21 (14.0)
Parietal	19 (12.7)
Cerebellum	16 (10.7)
Occipital	14 (9.3)
Temporal parietal	14 (9.3)
Temporal occipital	8 (5.3)
Insula	3 (2.0)
Spetzler-Martin grade	
I, No. (%)	15 (10.0)
II, No. (%)	56 (37.3)
III, No. (%)	52 (34.7)
IV, No. (%)	23 (15.3)
V, No. (%)	4 (2.7)
Associated nidal aneurysm	
Hemorrhage, No. (%)	16 (10.7)
Non-hemorrhage, No. (%)	2 (1.3)

### Mutational spectrum of gene related with HTT

According to previous studies in OMIM and literature, there are a total of four HHT-related genes included in our current study (Table 2). *ENG* encodes endoglin which is located on chromosome 9q and is associated with HHT1 (OMIM#187300); *ACVRL1* encodes activin receptor-like kinase type 1 (ALK1), which is located on chromosome 12q and is associated with HHT2 (OMIM#600376) [17, 18]. Decapentaplegic homolog 4 (*MADH4* or *SMAD4*) mutations can cause a syndrome which combines familial juvenile polyposis and HHT [20]. In addition, one study reported that *BMPR2* gene analysis is indicated in patients affected with both HHT and heritable pulmonary arterial hypertension (HPAH) [19]. We identified 5 patients with mutations in these genes in our sporadic BAVM database.

**Table 2:** Genes associated with syndromic brain arteriovenous malformation

Syndrome	Gene	Ref
Hereditary hemorrhagic telangiectasia	*ENG*	[17]
	*ACVRL1*	[18]
	*BMPR2*	[19]
	*SMAD4*	[20]
Capillary malformation-arteriovenous malformation	*RASA1*	[21]
Sturge-Weber syndrome	*GNAQ*	[22]

**Fig. 1:**
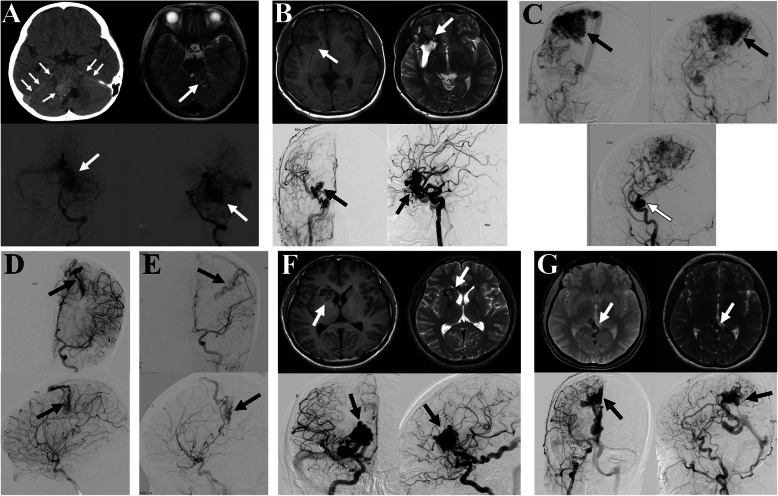
MRI, CT, and DSA imaging of patients carrying variants in syndromic genes. **A** Patient BAVM1 with *ENG* gene mutation c.901G>C. CT demonstrated subarachnoid hemorrhage in the distribution of posterior circulation, and DSA showed an AVM in the area of right cerebellar hemisphere. **B** Patient BAVM2 with ACVRL1 gene mutation c.1103C>T. MRI showed a hematoma and DSA showed an AVM in the right temporal lobe. **C** Patient BAVM3 with BMPR2 gene mutation c.775C>T. DSA revealed an AVM in the right side of the parietal lobe (black arrow) and an intracranial aneurysm in the internal carotid artery (white arrow). **D**, **E** Mutations of *BMPR2* c.2678G>A shared by BAVM4 and BAVM5. DSA showed an AVM in the left parietal lobe in two patients. **F**, **G** Patient BAVM6 and BAVM7with *RASA1* gene mutations c.1280G>A and c.3007G>A, respectively. MRI and DSA showed an AVM in the right basal ganglia and right parietal cerebral falx on two patients

There is one patient with an *ENG* mutation in our cohort. BAVM1 is a 10-year-old male presented with severe headache accompanied with nausea and vomiting. Computed tomography (CT) showed subarachnoid hemorrhage (SAH). MRI showed an AVM in the area of right cerebellar hemisphere, and DSA revealed a 70*25*25mm AVM with Spetzler-Martin Grade IV (Fig. 1A). The missense variant c.901G>C from BAVM1 was predicted to be deleterious (CADD=16.89). The variant is absent from the gnomAD database (Table 3), indicating that it is extremely rare in the general population.

**Table 3:** Summary of variants related to syndromes in syndromic brain arteriovenous malformation

Gene	Patient ID	Chr	Position	Zygosity	Mutation type	Variant nomenclature	V_R_:T_R_	CADD score	gnomAD_EAS_AF
*ENG*	BAVM1	9	130587169	Het	Missense	c.901G>C	39:73	16.89	0
*ACVRL1*	BAVM2	12	52309874	Het	Missense	c.1103C>T	38:65	29.2	0
*BMPR2*	BAVM3 BAVM4 BAVM5	2 2 2	203383698 203421066 203421066	Het Het Het	Missense Missense Missense	c.775C>T c.2678G>A c.2678G>A	39:65 47:99 47:99	31 24.4 24.4	0 0 0
*RASA1*	BAVM6 BAVM7	5 5	86649000 86685291	Het Het	Missense Missense	c.1280G>A c.3007G>A	38:72 28:67	25.6 25.3	0.0001095 0.00005439

*AF* allele frequency, *BAVM* brain arteriovenous malformation, *CADD* combined annotation-dependent depletion, *Chr* chromosome, *EAS* East Asian, *gnomAD* Genome Aggregation Database, *Het* heterozygous, *VR:TR* variant reads: total reads

A missense mutation c.1103C>T of *ACVRL1* is identified in another patient BAVM2. The bioinformatics tools predict that this mutation is damaging (CADD=29.2). It does not exist in gnomAD East Asian (EAS) database (Table 3). Patient BAVM2 is a 15-year-old female who presented with headache, vomiting, and epilepsy. MRI showed a hematoma in the right temporal lobe and DSA revealed a 40*15*10mm AVM with Spetzler-Martin Grade III (Fig. 1B).

Three patients have mutations in the *BMPR2* gene. The missense variant c.775C>T is absent from the gnomAD database (Table 3). Patient BAVM3 is a 23-year-old female presented with mild headache and epilepsy. DSA revealed a 60*45*30mm AVM with Spetzler-Martin Grade III in the right side of the parietal lobe and a intracranial aneurysm in the internal carotid artery (Fig. 1C). A research has shown that mutations of *BMPR2* can cause the Loeys-Dietz syndrome, which is an inherited connective tissue disorder can accompanied with aneurysms [23]. Two patients BAVM4 and BAVM5 have the same missense mutation (c.2678G>A). The absence of this variant in the gnomAD database and the enrichment of this variant in our cohort support the association between c.2678G>A and BAVM. Interestingly, both patients had arteriovenous malformations in the left parietal lobe (Fig. 1D, E), which further strengthened the evidence for the pathogenicity of variants in this gene in BAVM. Therefore, our findings may represent a phenotypic expansion for this gene.

### Mutational spectrum of gene related with CM-AVM

Capillary malformation-arteriovenous malformation (CM-AVM) syndrome is an autosomal dominant disorder due to germline heterozygous mutations in the *RASA1* gene [21] (Table 2). Some studies have observed that 3% of the malformations occurred in the cerebral. We identified two patients have the variant of *RASA1* gene in our database. They are both rare mutations (MAF<0.001) that are absent from the gnomAD database. CADD prediction indicated the mutations to be harmful (Table 3). BAVM6 is a 28-year-old male presented with severe headache accompanied with vomiting. DSA showed an AVM in the area of right basal ganglia (Fig. 1F). BAVM7 is a 27-year-old female presented with paroxysmal headache accompany with dizziness about 1 year, aggravating for 2 months. DSA showed a right parietal cerebral falx 28*30*25mm AVM belong to Spetzler-Martin Grade III (Fig. 1G). Our research indicates a more complex disease trait with potential digenic involvement in these patients.

### Sturge-Weber syndrome

Sturge-Weber syndrome is a rare neurovascular disorder associated with seizures, capillary malformation, cognitive impairments, and stroke-like episodes (SLE), arising from a somatic activating mutation in *GNAQ* [22] (Table 2). Brain arteriovenous malformations can be found in patients with Sturge-Weber syndrome [24]. We did not find any patients with *GNAQ* mutations in our database. Therefore, we did not identify correlation between the sporadic BAVM and Sturge-Weber syndrome, which might be associated with the limited sample size.

## Discussion

In this study, we utilized a WES dataset generated from a cohort of sporadic patients with BAVM. By inspecting genes associated with known vascular malformation syndromes, we found that deleterious variants in these genes are likely involved in the pathogenesis of sporadic BAVM. None of these variants have been reported in individuals with syndromic forms of BAVM or healthy individuals from public databases except two missense variants in *RASA1*.

The involvement of syndromic genes in sporadic cases have been reported in intellectual disability, which is difficult to diagnosis because of the absence of morphological clues and appropriate screening tools [25]. In a reported study, child-parent trios from ten centers in Switzerland and Germany were recruited. Of the 51 cases enrolled, 16 cases had de novo variants occurred in established intellectual disability-associated genes, including *STXBP1*, *SCN2A*, and *SYNGAP1* [25]. Interestingly, some patients did not manifest the expected syndromic conditions associated with their genetic variants, implicating a strong over-description of the present clinical syndromes [25], and an obscure boundary between syndromic and sporadic conditions. Another study reported a stop-gain variant Q829X in *OTOF*, which is associated with auditory neuropathy, in patients with prelingual non-syndromic hearing loss [26]. The authors investigated 28 independent trios in Spain and genotyped the subjects for microsatellite markers. After identifying the linkage of the disease to a certain genetic marker, they sequenced the surrounding genome regions and identified this novel variant in *OTOF*. This variant is the third most frequent variant which cause the prelingual deafness so far reported in the Spanish population [26].

The underlying mechanism underlying these findings may be the overlapping biological pathways perturbed in both syndromic and sporadic conditions. The pathogenesis of HHT is associated with the perturbation of BMP/TGF-β signaling pathway. TGF-β signaling is considered as crucial pathway in the physiological process of the vessels. Specifically, it has been shown to control proliferation, differentiation, apoptosis, migration, extracellular matrix (ECM) remodeling, immune functions, and tumor invasion/metastasis [27, 28]. Once TGF-β signaling activation occurs extracellularly, it is able to interact with and complex the type I and type II serine/threonine kinase receptors at the cell surface. During skeletal muscle development, the constitutively active type II receptor phosphorylates and activates the type I receptor, which in turn directly phosphorylates Smad3 to initiate signal transduction through the canonical cascades [29, 30]. In the non-canonical pathway, the type I receptor phosphorylates signal proteins that are involved in the activation of the mitogen-activated protein kinases (MAPKs), including non-canonical signaling through extracellular signal-related kinase (ERK), c-Jun *N*-terminal kinase (JNK), and p38 [31, 32]. Activated non-canonical signaling then regulate transcription factors and/or the Smad proteins through direct interactions or via downstream proteins to promote vascular differentiation, migration, and ECM remodeling [33]. Our results suggest that perturbation of BMP/TGF-β also underly the pathogenesis of isolated BAVM.

The pathogenesis of CM-AVM is associated with the Ras-Raf-MEK-ERK pathway. This pathway is initiated by an extracellular mitogen which binds to the receptor on the cell membrane, which allows the small GTPase Ras to swap the GDP for the GTP [34]. The activated Ras then activates the activity of protein kinase of the RAF kinase. Then, the RAF kinase activates MEK, i.e., MEK1 and MEK2 by phosphorylating them [35]. MEK then phosphorylates and activates the mitogen-activated protein kinase (MAPK). After that, MAPK regulates the activation of several transcriptional factors [36]. MAPK also controls the transcription of C-Fos gene and leads to changes in transcription of genes key for the cell cycle, which is important for the normal growth of the vascular system [37].

## Conclusions

In conclusion, we demonstrated that variants in syndromic vascular malformations play important roles in the etiology of sporadic BAVM. These may be due to the shared biological pathways between syndromic and sporadic forms of the disease.

## Data Availability

The datasets analyzed during the current study are available from the corresponding author on reasonable request.

## Abbreviations

BAVM: Brain arteriovenous malformation
MAPK: Mitogen-activated protein kinase
ERK: Extracellular signal-related kinase
JNK: c-Jun N-terminal kinase
ICH: Intracranial hemorrhage
HHT: Hereditary hemorrhagic telangiectasia
WES: Whole exome sequencing
CM-AVM: Capillary malformation-arteriovenous malformation
LoF: Loss of function
OMIM: Online Mendelian Inheritance in Man
MRI/MRA: Magnetic resonance imaging/angiography
CTA: Computed tomography angiography
DSA: Digital subtraction angiography
